# BMP-2 Promotes Oral Squamous Carcinoma Cell Invasion by Inducing CCL5 Release

**DOI:** 10.1371/journal.pone.0108170

**Published:** 2014-10-01

**Authors:** Mi-joo Kim, Kwang-mahn Kim, Jin Kim, Kyoung-nam Kim

**Affiliations:** 1 Department and Research Institute of Dental Biomaterials and Bioengineering, Dental Devices Testing & Evaluation Center, Brain Korea 21 Plus project, College of Dentistry, Yonsei University, Seoul, Republic of Korea; 2 Department of Oral Pathology, Oral Cancer Research Institute, Brain Korea 21 Plus project, College of Dentistry, Yonsei University, Seoul, Republic of Korea; Queen Mary University of London, United Kingdom

## Abstract

Bone morphogenetic protein-2 (BMP-2)-containing bone grafts are useful regenerative materials for oral and maxillofacial surgery; however, several *in* *vitro* and *in* *vivo* studies previously reported cancer progression-related adverse effects caused by BMP-2. In this study, by quantifying the rhBMP-2 content released from bone grafts, the rhBMP-2 concentration that did not show cytotoxicity in each cell line was determined and applied to the *in* *vitro* monoculture or coculture model in the invasion assay. Our results showed that 1 ng/ml rhBMP-2, while not affecting cancer cell viability, significantly increased the invasion ability of the cancer cells cocultured with fibroblasts. Cocultured medium with rhBMP-2 also contained increased levels of matrix metalloproteinases. rhBMP-2-treated cocultured fibroblasts did not show a prominent difference in mRNA expression profile. Some cytokines, however, were detected in the conditioned medium by a human cytokine antibody array. Among them, the cancer invasion-related factor CCL5 was quantified by ELISA. Interestingly, CCL5 neutralizing antibodies significantly reduced the invasion of oral cancer cells. In conclusion, our results suggest that 1 ng/ml rhBMP-2 may induce invasion of oral squamous cell carcinoma (OSCC) cells by CCL5 release in coculture models. Therefore, we propose that a careful clinical examination before the use of rhBMP-2-containing biomaterials is indispensable for using rhBMP-2 treatment to prevent cancer progression.

## Introduction

Oral cancer is the sixth most common malignancy worldwide and is occurring with increasing frequency [1]. The best known risk factors of this multifactorial disease include tobacco, alcohol, betel quid chewing, genetic predisposition, the presence of potentially malignant lesions, etc. [2], [3]. Inflammation caused by exogenous materials is also known to be associated with cancer initiation and development. Some biomaterials used for the recovery of human body during surgery and healing processes were shown to be irritants or oncogenes. Especially, in dentistry, it is known that dental implants for hard tissue reconstruction can be causative agents of unexpected side effects including oral cancer in some cases [4]–[6].

Currently, bone morphogenetic protein-2 (BMP-2) has become a popular commercial drug used for hard tissue regeneration in orthopedic surgery and dental treatment in the form of bone graft coating, membranes, or solutions. BMPs are originally identified as osteogenic cytokines that promote bone and cartilage formation *in* *vivo* [7], [8]. BMPs regulate developmental epithelial-to-mesenchymal transition, including neural crest migration and cardiac morphogenesis [9]. However, BMP-2 overexpression has been demonstrated to result in changes in cell morphology and increased cell motility and invasiveness in the pancreatic, gastric, prostate and breast cancer cells [10]–[14]. Moreover, a recent report published by the Food and Drug Administration described BMP-2-induced adverse effects such as bone resorption, local inflammation, ectopic bone formation, and cancer; specifically, 86 reports of oral and maxillofacial surgery from 2002 to 2011 were found to display negative outcomes [15]. Therefore, it is inevitable to establish methods that could accurately evaluate whether BMP-2 exogenously injected for oral hard tissue regeneration is associated with oral cancer.

The relationship between cancer cells and BMP-2 has been investigated in several studies. During clinical research, spinal surgery with high-dose rhBMP-2 treatment led to four-fold increase in the number of new malignancies after 24 months, compared to the untreated group, and the discrepancy increased after 60 months [16]. Studies analyzing 25 tongue squamous cell carcinomas by gene microarray and qPCR analyses revealed that BMP-2 gene expression may be associated with lymph node metastasis [17]. In addition, ectopic expression of BMP-2 stimulated angiogenesis in developing tumors and resulted in significantly increased local invasion and metastasis in melanoma [18], [19], lung cancer cells [20], [21], and breast cancer cells [22]. However, findings of these previous studies are insufficient for revealing the role of BMP-2 and its mechanism related to oral cancer progression.

The effect of fibroblasts on oral cancer progression related to BMP-2 has not yet been considered, although stromal fibroblasts are known as important and main host cell types in tumor microenvironments. Cancer-associated fibroblasts are responsible for the production of paracrine growth factors, proteolytic enzymes, and extracellular matrix (ECM) components, through which the combination of epithelial cancer cells and fibroblasts promote tumorigenesis and development of tumors that are larger than malignant epithelia alone [23]. Based on these findings, investigators should consider a combined evaluation system with regard to cancer cells and nearby stroma, especially fibroblasts, and design *in* *vitro* experimental models that could reflect these *in* *vivo* environments. For this experimental design, there were some invasion assays in the previous studies [24]–[26], but more natural methods that enable an easy analysis and quantification of OSCC invasion *in* *vitro* should be used for accurate evaluations of cancer progression.

Biological reactions in response to various irritants are different, depending on the general conditions and local status of hosts. Therefore, in this study, we assumed the situation that clinicians would not detect or disregard the signs of pre-existing oral cancer cells before BMP-2 treatment for bone-defective areas. Considering this clinical situation, we developed an *in* *vitro* system that combined BMP-2 and pre-existing oral cancer cells with or without fibroblasts. With the use of cancer cells either monocultured or cocultured with fibroblasts *in* *vitro*, we studied the effects of BMP-2 on the invasion ability of oral squamous cell carcinoma (OSCC) cell lines and analyzed the transformation and releasing factors of these cells. Our results suggest that detection of premalignant signs is important prior to BMP-2 treatment and that clinicians should recognize the possible danger of BMP-2 depending on patients’ oral conditions. Furthermore, this evaluation method for regenerative materials or drugs would be useful in the development and approval process.

## Materials and Methods

### Quantification of BMP-2 extracted from bone graft materials by ELISA

Each bone graft material (2 g) described in Table S1 was incubated with 2 ml of complete protease inhibitor cocktail (Roche, Mannheim, Germany) at 4°C for 4 weeks. After extraction, samples were centrifuged at 12,000 rpm for 10 min, and the supernatants were analyzed using the BMP-2 ELISA Kit (R&D Systems, Minneapolis, MN, USA) [28], [29]. This experiment was repeated independently three times under the same conditions.

### Preparation of OSCC cell lines, immortalized fibroblasts, and rhBMP-2

Two OSCC cell lines, namely, YD-10B and YD-38 (origin, human tongue and human lower gingiva, respectively), were obtained from the Korean Cell Line Bank (Seoul, Korea) and cultured as described previously before [30]. HSC-2 cells (origin, human tongue), a gift from Prof. Takashi Muramatsu, Tokyo Dental College, Japan, were maintained in DMEM: Ham’s F12 (3∶1; Gibco, NY, USA), supplemented with 10% FBS and 1% penicillin/streptomycin. Human immortalized fibroblasts (hTERT-hNOF), which were established previously [31], were cultured either alone or with OSCC cell lines. rhBMP-2 was purchased from the Korea Bone Bank (Seoul, Korea) and diluted in cell culture medium to various concentrations.

### Cell viability assay

Three OSCC cell lines (YD-10B, YD-38, and HSC-2) and fibroblasts (2×10^4^ cells/well) were seeded on 24-well culture plates and cultured for 24 h. Next, cells were treated with rhBMP-2 (0, 1, 5, 10, 20, and 40 ng/ml) in serum-free medium for 24, 48, or 72 h under 5% CO_2_. The rhBMP-2 non-treated group was used as a control. The cell viability assay was performed using WST Solution (Daeil Lab, Seoul, Korea) according to the manufacturer’s instructions, and the absorbance was measured at 450 nm by an Epoch spectrophotometer (BioTek, VT, USA). The cell viability of each experimental group was expressed as the percentage of the cell viability of the control. Three independent experiments were performed under identical conditions.

### Invasion assay

To evaluate the invasive activity of cancer cells, a modified transwell invasion assay was carried out as described previously [32]. Briefly, 24-well transwell plates (Corning, NY, USA), along with inserts containing 8-µm pore size filters coated with collagen type I (30 µl/well), were used. Fibroblasts (2×10^4^ cells/well) were placed in the lower wells of chambers with 10% FBS and 1% antibiotics. After their attachment for 24 h, media for fibroblasts was replaced with serum-free one containing rhBMP-2 (1 ng/ml). And each OSCC line (2×10^4^ cells/transwell) was seeded in the upper part (porous filter with collagen layer). OSCC monocultures were obtained by culturing OSCC cell lines without fibroblasts on the same side of filters. The rhBMP-2 non-treated group was used as a control. This condition was maintained for 48 h. OSCC cells penetrating though filters were fixed with formaldehyde (Sigma-Aldrich, MO, USA), stained with hematoxylin solution (Sigma-Aldrich), and counted under a light microscope. Experiments were repeated three times under identical conditions.

### Microarray analysis

OSCC cells (YD-38 or HSC-2) and fibroblasts (1.7×10^5^) were seeded in upper and lower chambers, respectively, of 6-well transwell plates containing collagen-coated transmembrane filters (pore size, 1 µm) (Becton, Dickinson and Company, Franklin Lakes, NJ). After cells were incubated either with or without rhBMP-2 (1 ng/ml) in serum-free medium for 48 h, total RNA was isolated from underlying fibroblasts by using Trizol Reagent (Invitrogen, Carlsbad, CA) and subjected to microarray analysis, as described in the Methods S1.

### Human matrix metalloproteinase (MMP) antibody array

Cell supernatants were obtained from the cocultures of YD-38 and fibroblasts or monocultured fibroblasts treated with or without rhBMP-2 (1 ng/ml) for 48 h and subjected to the human MMP antibody array (Ray Biotech, GA, USA). The relative expression levels of released MMPs and tissue inhibitors of metalloproteinases (TIMPs) were calculated by comparing their levels before and after rhBMP-2 treatment.

### Measurement of secreted cytokines by the human cytokine antibody assay and ELISA

YD-38 cells cocultured with fibroblasts in 24-well transwell plates were treated with or without rhBMP-2 (1 ng/ml) for 48 h, and the supernatants were then subjected to the human cytokine antibody array (Ray Biotech, GA, USA). The relative expression level of cytokines was determined by comparing signal intensities.

Monoculture supernatants were obtained by culturing OSCC cell lines or fibroblasts on the same side of filters as described in the coculture design. Cells were cultured for 48 h with either 1 ng/ml rhBMP-2-containing medium or pure serum-free medium. The CCL5 levels in supernatants obtained from monoculture or coculture models were quantified using the Human CCL5 ELISA Kit (R&D Systems, MN, USA). Experiments were performed three times under identical conditions.

### Treatment of coculture models with CCL5 neutralizing antibodies and rhBMP-2 and invasion assay

CCL5 neutralizing antibodies (R&D Systems, MN, USA), along with rhBMP-2, were added to coculture models in 24-well transwell plates coated with collagen type I (50 µl/well) for 48 h. Control group was set as the one with rhBMP-2 (1 ng/ml) and IgG antibody. Three treatment groups were as follows: control, rhBMP-2 (1 ng/ml) plus CCL5 neutralizing antibody (0.01 µg/ml), and rhBMP-2 (1 ng/ml) plus CCL5 neutralizing antibody (0.05 µg/ml). Finally, invading cells were fixed, stained, and counted as described before.

### Statistical analysis

Statistical comparisons were performed by the two-sample *t* test. Data were given as means ± standard deviations in graphs. Significance was established when P<0.05.

## Results

### BMP-2 content in bone graft materials

As shown in Table S1, the synthetic rhBMP-2-coated product BMP (Cowellmedi, Korea) released the highest amount of BMP-2. The BMP-2 content in the allografts, xenografts, and synthetic grafts varied and did not depend on the type of bone grafts. The average concentration of rhBMP-2 extracted from all graft materials was 1047 pg/ml.

### rhBMP-2 (1 ng/ml) has no significant effect on the cell viability of three OSCC cell lines and fibroblasts

**Figure 1:**
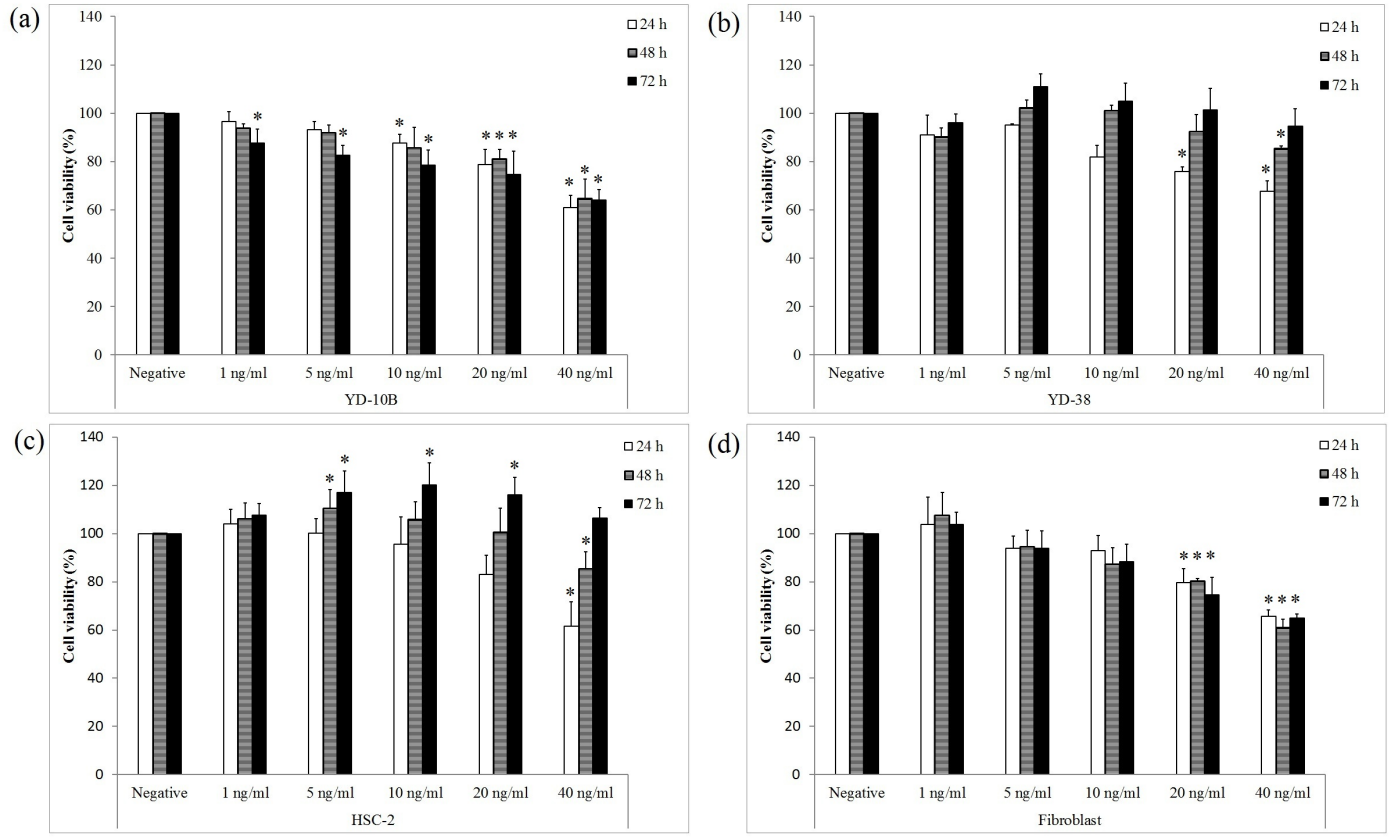
Cell viability of three OSCC cell lines and fibroblasts. Data are expressed as the percentage of respective non-treated control groups. Data shown are means of three independent repeats, and error bars indicate standard deviation. *P<0.05 compared to the negative control.

While the cell viability of three OSCC cell lines and fibroblasts was decreased by rhBMP-2 in a concentration-dependent manner (0–40 ng/ml; Fig. 1), 1 ng/ml rhBMP-2 did not reduce cell viability, except in case of YD-10B that were treated for 72 h. Notably, in contrast to YD-10B and fibroblasts, the viability of YD-38 and HSC-2 cells increased at all concentrations after 48 and 72 h of treatment. Since 1 ng/ml rhBMP-2 had no significant effects on the cell viability of three OSCC cell lines and fibroblasts after 24 and 72 h h and the average rhBMP-2 concentration extracted from graft materials was 1047 pg/ml, rhBMP-2 at the concentration of 1 ng/ml was used in subsequent experiments.

### rhBMP-2 promotes cell invasion of three OSCC cell lines

**Figure 2:**
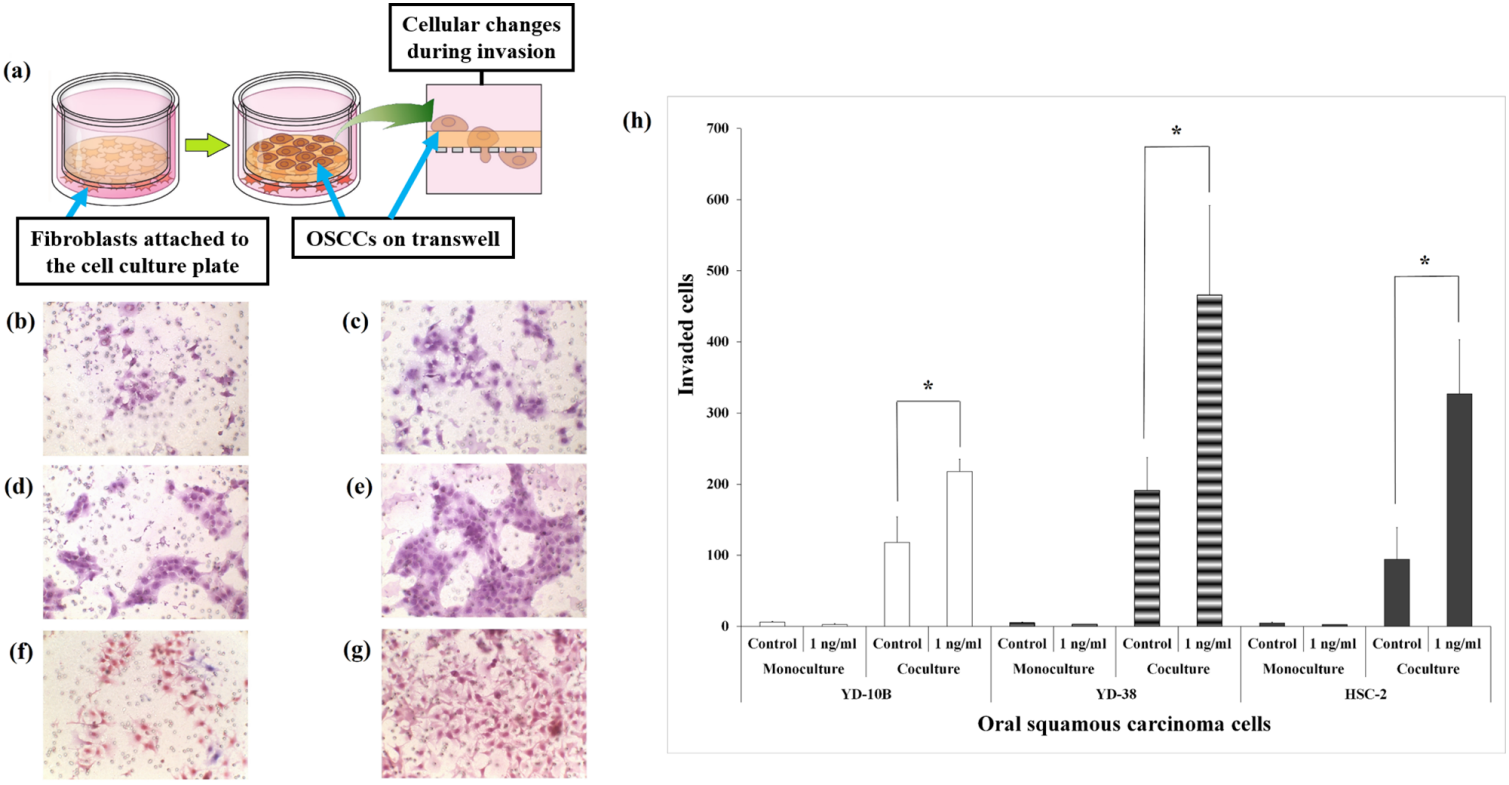
Invasion assay in monoculture or coculture models. (a) Schematic diagram of the invasion assay and cellular changes. Carcinoma cells on the transwell penetrate the collagen membrane when cocultured with fibroblasts and then finally attach to the lower surface of the transwell. (b–g) Cocultured YD-10B (b and c), YD-38 (d and e), and HSC-2 (f and g) cells either untreated (b, d, and f) or treated with 1 ng/ml rhBMP-2 (c, e, and g). (h) Quantification of the invasion ability of OSCC cells. Data shown are means of three independent repeats, and error bars indicate standard deviation. *P<0.05 compared to the control.

The schematic diagram of the invasion assay is shown in Fig. 2a. Monocultured OSCC cell lines did not invade through collagen membranes, regardless of rhBMP-2 treatment (Fig. 2h, all mean values were lower than 10). In contrast, YD-10B, YD-38, and HSC-2 cocultured with fibroblasts invaded to the bottom surface of collagen-coated transwell plates at significantly higher levels without FBS (Fig. 2h). Especially, upon rhBMP-2 treatment, cocultured YD-38 proliferated into clusters and showed the highest number of invading cells among all groups (mean; 466) (Fig. 2d vs. 2e).

### rhBMP-2 causes little genetic changes in cocultured fibroblasts

The fibroblasts cocultured with YD-38 or HSC-2 were treated with 1 ng/ml rhBMP-2, and genome-wide gene expression profiles of the fibroblasts were then analyzed by the microarray analysis. Significant genetic changes were only detected in the FAM21A gene (fold change, ∼2.2; P<0.05; Table S2). Alterations with over 1.5-fold changes were also summarized in Table S2.

### rhBMP-2 increases MMP release in coculture models

**Figure 3:**
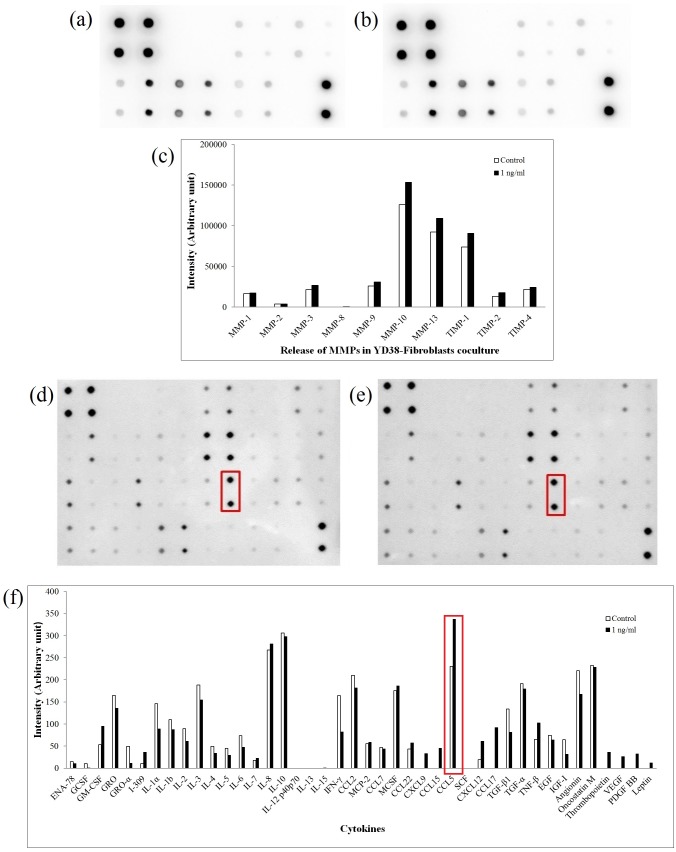
MMP and cytokine antibody array for cocultured YD-38 and fibroblasts. (a–c) MMP array for cocultured YD-38 cells either untreated (a) or treated with rhBMP-2 (b). (c) Quantification of the levels of various MMPs. (d–f) Cytokine array of cocultured YD-38 cells either untreated (d) or treated with rhBMP-2 (e). (f) Quantification of the levels of various cytokines. Red boxes in (d) and (e) show the changes of signal intensities of CCL5 before and after 1 ng/ml rhBMP-2 treatment, respectively, which were quantified and compared in the red box of (f).

The release of MMPs from monocultured fibroblasts was found to be decreased by treatment with 1 ng/ml rhBMP-2 when compared with the non-treated control (Fig. S1). However, the coculture supernatant of YD-38 and fibroblasts showed increased levels of all MMPs, especially MMP-10, MMP-13, and TIMP-1, after rhBMP-2 treatment, compared to the non-treated control (Fig. 3a–c).

### Treatment of cocultured OSCC models with rhBMP-2 significantly increases CCL5 release

The human cytokine array analysis indicates that the fibroblasts cocultured with YD-38 and treated with 1 ng/ml rhBMP-2 showed decreases in the release of most cytokines, except CCL5, CXCL12, CCL17, VEGF, and PDGF (Fig. 3f). The level of CCL5, in particular, was markedly increased in intensity from 230 to 337 (Fig. 3d–f, in the red box). Since CCL5 was previously shown to be a representative protein related to cancer invasion [33]–[36], we considered that CCL5 may be a key factor in oral cancer invasion upon rhBMP-2 treatment.

**Figure 4:**
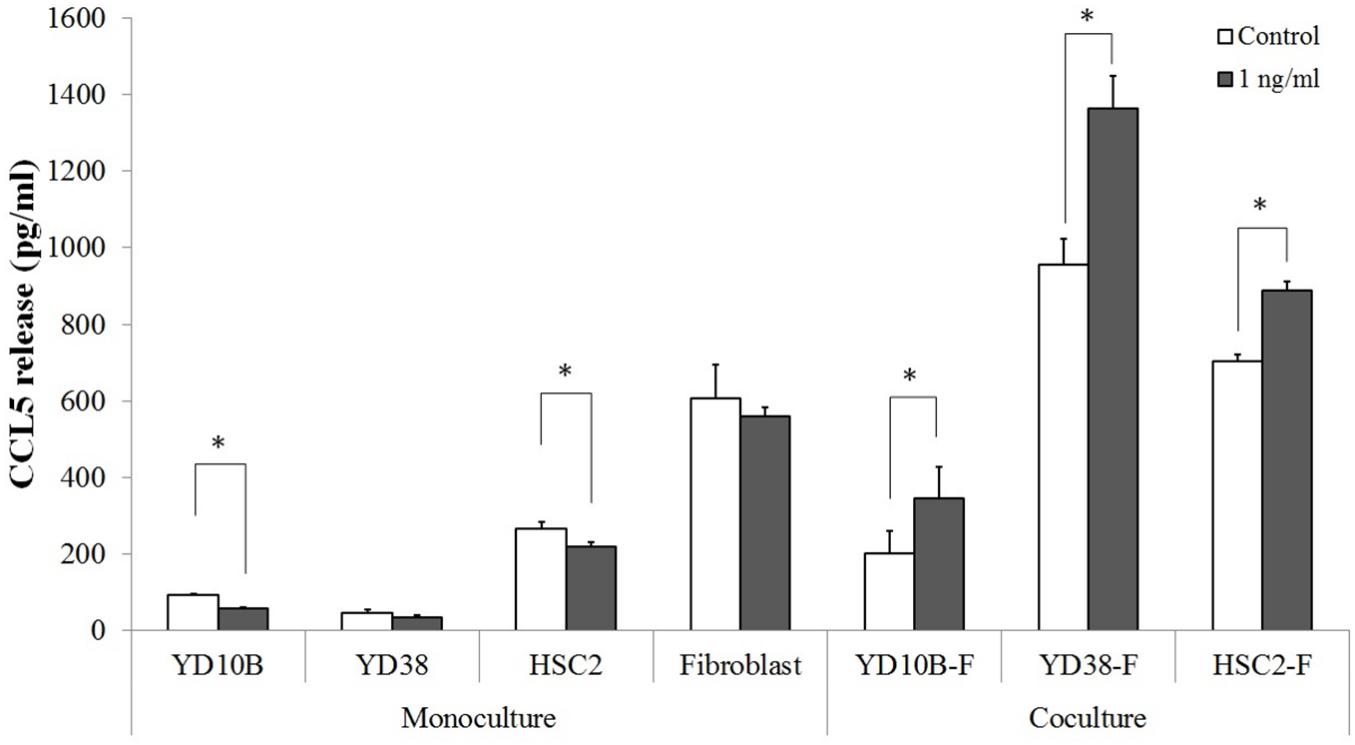
Human CCL5 ELISA of monocultured or cocultured medium with or without 1 ng/ml rhBMP-2. Data shown are means of three independent repeats, and error bars indicate standard deviation. *P<0.05 compared to the control.

The release of human CCL5 proteins was further validated by ELISA (Fig. 4). In the coculture groups (YD10B-F, YD38-F, and HSC2-F), treatment with 1 ng/ml rhBMP-2 resulted in significant increase in CCL5 release (P<0.05); in contrast, monocultured OSCC cell lines or fibroblasts showed either reduced or unchanged levels of CCL5 release after rhBMP-2 treatment.

### CCL5 neutralizing antibodies inhibit OSCC cell invasion induced by rhBMP-2

**Figure 5:**
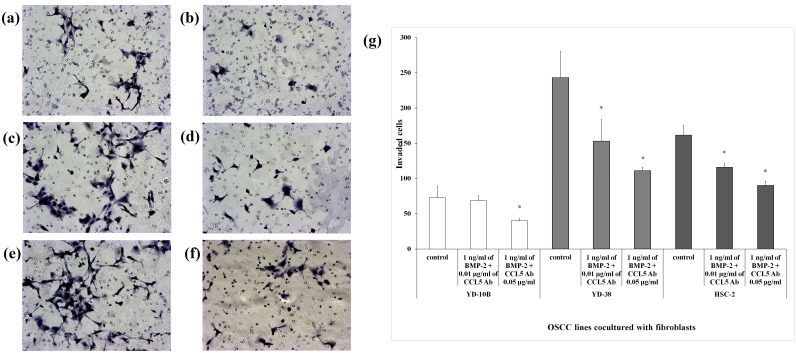
Invasion assay of coculture models treated with rhBMP-2 and CCL5 neutralizing antibodies. (a–f) Representative invasion results of YD-10B (a and b), YD-38 (c and d), and HSC-2 (e and f) cocultured cells treated with either 1 ng/ml rhBMP-2 only (a, c, and e) or both rhBMP-2 and 0.05 µg/ml CCL5 neutralizing antibodies (b, d, and f). (g) Quantification of the invasion ability of OSCC cell lines. Data shown are means of three independent repeats, and error bars indicate standard deviation. *P<0.05 compared to the control.

As shown in Fig. 2, rhBMP-2 (1 ng/ml) treatment led to significant increases in cell invasion of three OSCC cell lines; however, the number of OSCC cells penetrating through 50 µl of collagen type I were less than those penetrating through 30 µl of collagen (Fig. 5). As shown in Fig. 5g, 0.01 µg/ml of CCL5 neutralizing antibodies reduced the amount of invading cells in every group, compared to the control (YD-10B; 73 to 69, YD-38; 243 to 153, HSC-2; 162 to 116). In particular, in YD-10B and YD-38 cells, the effect of CCL5 neutralizing antibodies was notably greater at the concentration of 0.05 µg/ml than at 0.01 µg/ml.

## Discussion

Multiple microenvironmental alterations occur during cancer progression; these changes include increased numbers of fibroblasts, blood vessels, immune cells, and ECM components. The crosstalk between normal fibroblasts and cancer-associated fibroblasts and epithelial cells is also known to contribute to cancer progression. Thus, there are modifications of normal fibroblasts to cancer fibroblasts and remodeling of ECM by degradation and increased stiffness. The coculture condition used in this study has an advantage for evaluating cell-to-cell interactions in cancer microenvironments, as well as the contribution of stromal fibroblasts to cancer cells. We believe that such an *in* *vitro* model (cocultures of OSCC cell lines and fibroblasts) mimics the *in* *vivo* system and can therefore be a critical and powerful tool. In a previous study, cocultures of breast carcinoma cell lines with fibroblastic stromal cells resulted in marked induction of aromatase enzymatic activity and mRNA expression [37], both of which are generally below the detection level partly due to the lack of interactions between parenchymal or carcinoma cells and stromal cells. Therefore, in this report, we evaluated the effects of rhBMP-2 on cancer cells in coculture models. Our results show that while monocultured cancer cells did not exhibit any cell invasion ability, all cocultured ones showed increased invasion rates (Fig. 2h).

rhBMP-2 exerted inhibitory effects on the growth of OSCC cell lines and fibroblasts and these effects became more evident as the rhBMP-2 concentration was increased from 0 to 40 ng/ml, as shown in Fig. 1. These results are in agreement with those of previous studies on the effects of rhBMP-2 on HUVEC cells, breast tumor cells, gastric mucosal cells, and gastric cancer cells [38]–[40]. Specifically, BMP-2 was shown to not only cause G1 arrest of monocultured cancer cells but also reduce the number of cells in S phase and their CDK4 expression [39]. This study also proved that rhBMP-2 exerted similar effects on the OSCC cell lines and fibroblasts. Importantly, rhBMP-2 at the concentration of 1 ng/ml, which is approximately the average content of rhBMP-2 released from bone graft materials (Table S1), did not affect the cell viability (Fig. 1). Therefore, 1 ng/ml was considered to be an appropriate concentration used to evaluate the effects of rhBMP-2 on cancer cells in subsequent experiments, although the content of BMP-2 obtained from bone graft materials depends on the extraction method and time and dilution ratios.

As shown in Fig. 2 and 5, 1 ng/ml rhBMP-2-treated coculture models increased cancer cell invasion through 30 or 50 µl of collagen membranes. In a previous study, BMP2 increased the invasion ability of OSCC cell lines (demonstrating gene expression of BMP2) and this effect enlarged when BMP2 combined with FBS was treated to the cells. OSCC cell lines cocultured with fibroblasts can release chemokines and cytokines to the media and invade through transwell without FBS [41]. It was known that cancer cell-derived TGF-β1 was responsible for inducing trans-differentiation of oral normal fibroblasts into cancer associated fibroblasts (CAF)-like cells and CAF-like cells secret high levels of growth factors that enhance OSCC cell line invasion and proliferation [42]. Therefore, the existence of fibroblasts and its stimulation have a key role in increased invasion ability of OSCC cell lines by rhBMP-2 in this study.

Increased OSCC cell invasion occurred concurrently with the increased levels of matrix metalloproteinases (MMPs) and their endogenous inhibitors, namely, tissue inhibitors of metalloproteinase (TIMPs). It was already known that increased expression of certain MMPs plays a significant role in ECM degradation, disrupts cell-cell contact through E-cadherin [43], and therefore correlates with tumor invasion [44]. Among the upregulated MMPs shown in Fig. 3, MMP-10 has been suggested to play an important role in the invasion and metastasis of head and neck cancer [45], and MMP-13 is known as a commonly upregulated gene by cancer invasion-related factors, thereby promoting tumor angiogenesis [46]. TIMP-1 is frequently overexpressed in several types of human cancers and serves as a prognostic marker [47], [48]. Taken together, our data support that rhBMP-2 increases the invasion ability of cancer cells.

CCL5 level increased markedly after the treatment of 1 ng/ml rhBMP-2 in the YD-38 coculture model. Since this cytokine has been previously suggested to be a factor related to cancer cell invasion, we next investigated the role of CCL5 in the cancer cell invasion assay. In Fig. 5, YD-10B, YD-38, and HSC-2 coculture models that were treated with both rhBMP-2 and CCL5 antibodies had less invading cells, compared to those treated with rhBMP-2 alone (P<0.05), and that the inhibitory effects were dependent on the concentration of the neutralizing antibodies. These data clearly suggest that interfering with CCL5 signaling by using specific neutralizing antibodies may be an attractive approach to offer protection against OSCC cell invasion. The investigation by Salo S et al. also showed such results in human squamous cell carcinoma originated from tongue, in which CCL5 function-blocking monoclonal antibody inhibited the invasion bone marrow-derived multipotent mesenchymal stromal cells (BMMSCs)-promoted HSC-3 cell invasion area [27]. Similar results have also been shown in colorectal cancer, breast cancer, and prostate cancer [49]–[51]. However, noticeably, interfering with CCL5 signaling did not completely inhibit rhBMP-2-induced cell invasion, implicating the involvement of other pathways. For instance, CXCL9 [51], CXCL12 [52], PDGF-BB [53], and VEGF [54], all of which are well-known factors associated with cancer cell invasion, were also detected by the cytokine antibody array in this study. Future studies will be necessary to determine whether combined blockage of these proteins will provide protection against BMP-2-induced oral cancer progression.

Based on the invasion assay, the cocultured OSCC cell lines treated with rhBMP-2 were found to exhibit more invasive characteristics *in* *vitro* than the monocultured ones. However, the fibroblasts cocultured with YD-38 or HSC-2 demonstrated no common genetic alterations with over 2-fold changes. Although one common gene, RN7SK, was found to be downregulated (fold change, ∼1.5) in Table S2, its expression was comparatively weak. A better understanding of the relationship of RN7SK with cytokines and cancer cell invasion will be necessary for elucidating its correlation with rhBMP-2 and cancer invasion.

Despite these limitations, our study discusses the probability of unconsidered adverse effects caused by 1 ng/ml rhBMP-2 and warns rhBMP-2-induced invasion and progression of pre-existing cancer cells that might not be detected in a diagnostic process. In addition, this study also emphasizes the importance of the *in* *vitro* coculture system (cancer cells with fibroblasts) for the evaluation of cancer progression induced by regenerative biomaterials. However, it is too early to conclude that rhBMP-2 is a carcinogenic material at specific concentrations because many previous studies evaluated the effects of rhBMP-2 *in* *vivo*. Therefore, generation of an appropriate animal model for the application of rhBMP-2 is additionally necessary in a further study. In conclusion, considering the chances of negative effects with oral usage of rhBMP-2, close examination and thorough diagnosis of cancers are indispensable before the use of rhBMP-2. Additionally, malignant tissues near the defective areas should be treated or removed in advance to eliminate the possibility of adverse effects caused by rhBMP-2.

## Supporting Information

Figure S1 **MMP array analysis with or without 1** **ng/ml rhBMP-2 treatment.** Monocultured fibroblasts either untreated (a) or treated with rhBMP-2 (b). (c) Quantification of the levels of various MMPs.
(JPG)

Table S1 **rhBMP-2 content in bone grafts.**
(JPG)

Table S2 **Genetic changes of fibroblast cocultured with YD-38 (a) or HSC-2 (b) after rhBMP-2 treatment by microarray assay.**
(JPG)

Methods S1 **Microarray analysis.**
(DOC)
