# Supplementary figures and images for: BMP-2 Promotes Oral Squamous Carcinoma Cell Invasion by Inducing CCL5 Release
Source: PLoS One. 2014 Oct 1;9(10):e108170. doi: 10.1371/journal.pone.0108170 (PMC4182698; doi:10.1371/journal.pone.0108170)

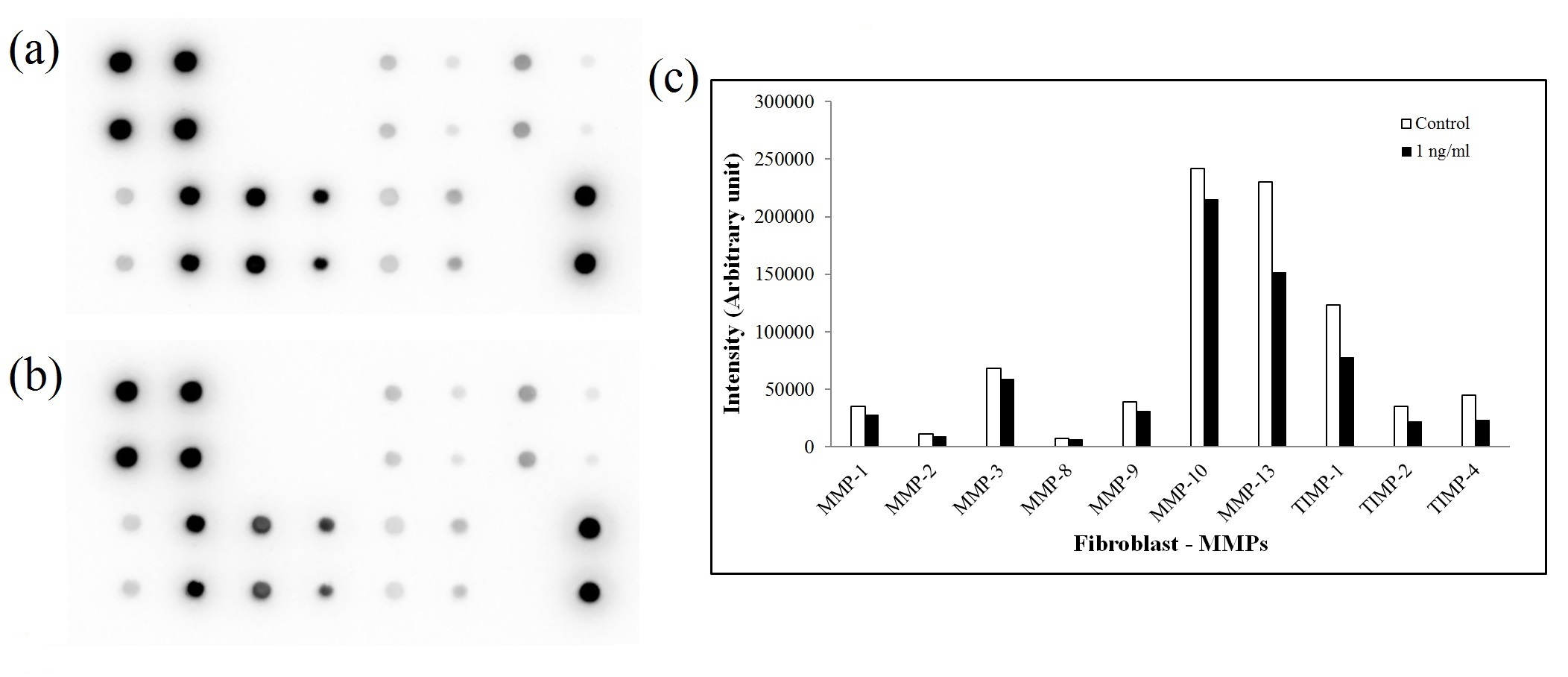

Supplement: Figure S1 — MMP array analysis with or without 1 ng/ml rhBMP-2 treatment. Monocultured fibroblasts either untreated (a) or treated with rhBMP-2 (b). (c) Quantification of the levels of various MMPs. (JPG) [file pone.0108170.s001.jpg]

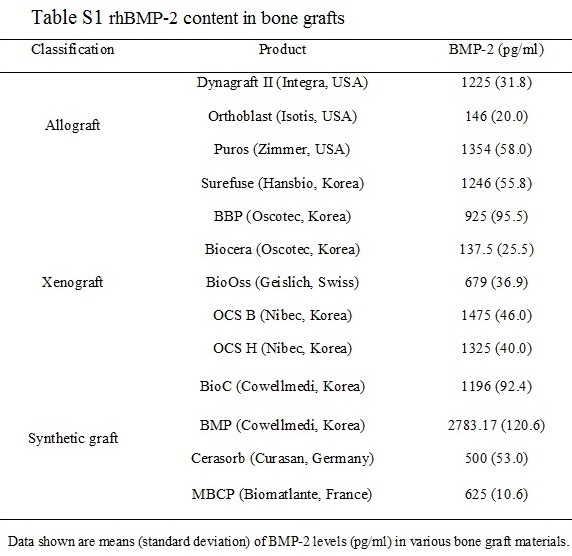

Supplement: Table S1 — rhBMP-2 content in bone grafts. (JPG) [file pone.0108170.s002.jpg]

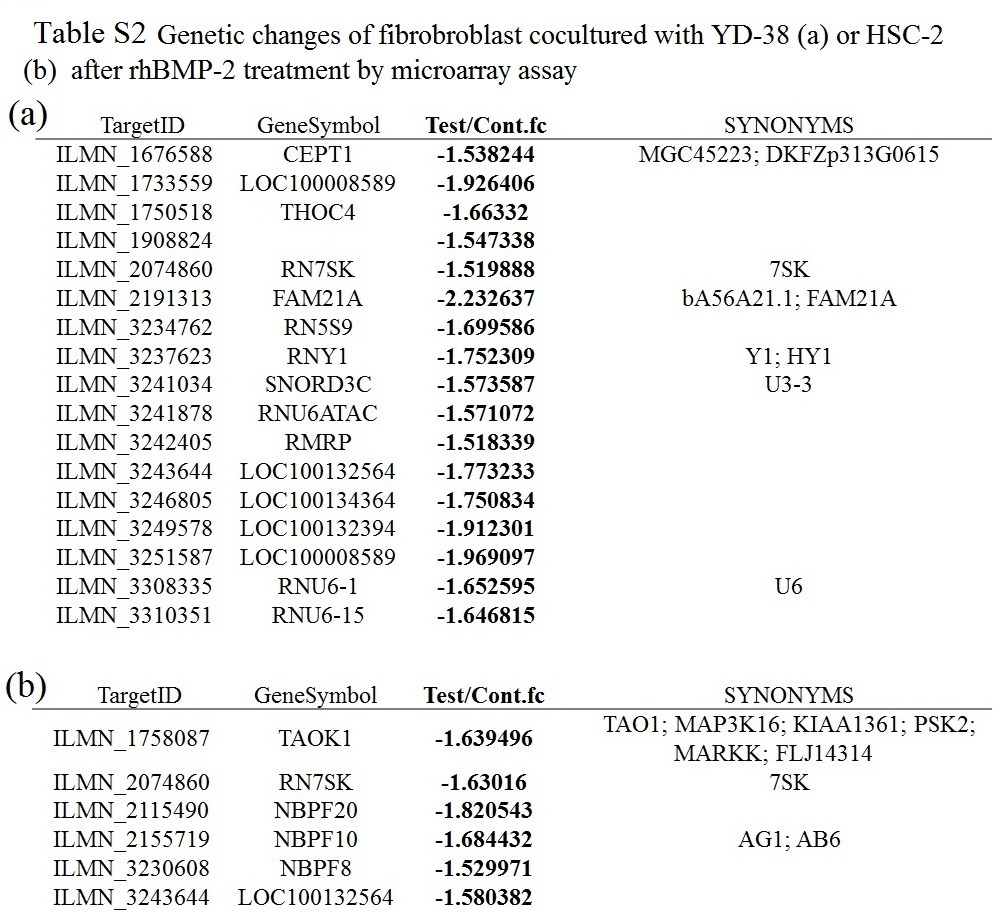

Supplement: Table S2 — Genetic changes of fibroblast cocultured with YD-38 (a) or HSC-2 (b) after rhBMP-2 treatment by microarray assay. (JPG) [file pone.0108170.s003.jpg]
