# Supplementary material for: BMP-2 Promotes Oral Squamous Carcinoma Cell Invasion by Inducing CCL5 Release
Source: PLoS One. 2014 Oct 1;9(10):e108170. doi: 10.1371/journal.pone.0108170 (PMC4182698; doi:10.1371/journal.pone.0108170)
Supplement: Methods S1 — Microarray analysis. (DOC) [file pone.0108170.s004.doc]

**Methods S1**

*Microarray analysis*

*(1) Preparation of fluorescent DNA probe and hybridization*

Total RNA was extracted from the human cancer cell line using the TRI REAGENT (MRC, OH) according to the manufacturer’s instructions. Each total RNA sample (30 μg) was labeled with Cyanine (Cy3) or Cyanine (Cy5) conjugated dCTP (Amersharm, Piscataway, NJ) by a reverse transcription reaction using reverse transcriptase, SuperScrip ll (Invitrogen, Carlsbad, California). The labeled cDNA mixture was then concentrated using ethanol precipitation method. The concentrated Cy3 and Cy5 labeled cDNAs were resuspended in 30 μL of hybridization solution (GenoCheck, Korea). After two labeled cDNAs were mixed, placed on OpArray Human genome 35K (OPHSV4, Operon Biotechnologies, GmbH) and covered by a MAUI FL chamber (Biomicro systems, Inc. UT). The slides were hybridized for 12 h at 62 °C MAUI system (Biomicro systems, Inc. UT). The hybridized slides were washed in 2×SSC, 0.1 % SDS for 2 min, 1×SSC for 3 min, and then 0.2×SSC for 2 min at room temperature. The slides were centrifuged at 3000 rpm for 20 sec to dry.

*(2) Data analysis*

Hybridized slides were scanned with the Axon Instruments GenePix 4000B scanner and the scanned images were analyzed with the software program GenePix Pro 5.1 (Axon, CA) and GeneSpring GX 7.3.1 (Agilent Technologies, CA). Spots that were judged as substandard by visual examination of each slide were flagged and excluded from further analysis. Spots that had dust artifacts or spatial defects were manually flagged and excluded. To filter out the unreliable data, spots with signal-to-noise (signal – background - background SD) below 10 were not included in the data. Data were normalized by Global, lowess, print-tip and scaled normalization for data reliability. Fold change filters included the requirement that the genes be present in at least 200% of controls (fold change: ≥ 2.0) for up-regulated genes and lower than 50% of controls (fold change: ≤ 0.5) for down-regulated genes. Data were clustered groups of genes that behave similarly across a time course experiments using GeneSpring GX 7.3.1(Agilent Technologies, CA). We used an algorithm, based on the Euclidian distance, to separate the gene of similar patterns.
